# Smokers' reactions to FDA regulation of tobacco products: Findings from the 2009 ITC United States survey

**DOI:** 10.1186/1471-2458-11-941

**Published:** 2011-12-16

**Authors:** Brian V Fix, Richard J O'Connor, Geoffrey T Fong, Ron Borland, K M Cummings, Andrew Hyland

**Affiliations:** 1 Department of Health Behavior, Roswell Park Cancer Institute, Elm and Carlton Street, Buffalo, NY 14263, USA; 2 Department of Psychology, University of Waterloo, Waterloo, ON, N2L 3G1, Canada; 3 Ontario Institute for Cancer Research, Toronto, ON, M5G 1L7, Canada; 4 The Cancer Council Victoria, Carlton, VIC, 3053, Australia; 5 Department of Psychiatry & Behavioral Sciences, Medical University of South Carolina, Charleston, SC, USA

## Abstract

**Background:**

On June 22, 2009, the US FDA was granted the authority to regulate tobacco products through the Family Smoking Prevention and Tobacco Control Act (FSPTCA). The intent is to improve public health through regulations on tobacco product marketing and tobacco products themselves. This manuscript reports baseline data on smokers' attitudes and beliefs on specific issues relevant to the FSPTCA.

**Method:**

Between November 2009 and January 2010, a telephone survey among a nationally representative sample of n = 678 smokers in the US was performed as part of the International Tobacco Control (ITC) United States Survey. Participants answered a battery of questions on their attitudes and beliefs about aspects of the FSPTCA.

**Results:**

Most smokers were unaware of the new FDA tobacco regulations. Smokers indicated support for banning cigarette promotion and nearly a quarter supported requiring tobacco companies to sell cigarettes in plain packaging. Seventy two percent of smokers supported reducing nicotine levels to make cigarettes less addictive if nicotine was made easily available in non-cigarette form.

**Conclusion:**

Most smokers were limited in their understanding of efforts to regulate tobacco products in general. Smokers were supportive of efforts to better inform the public about health risks, restrict advertising, and make tobacco products less addictive.

## Background

On June 22, 2009, the United States Food and Drug Administration (FDA), through the Family Smoking Prevention and Tobacco Control Act (FSPTCA), [1] was granted the authority to regulate the manufacturing, marketing, and sale of tobacco products with a mandate to reduce tobacco-related morbidity and mortality.

Under the FSPTCA, the FDA has the authority to set performance standards on specific constituents of tobacco or tobacco smoke for the benefit of public health (with the exception of completely eliminating nicotine) [1], ban additives that are used to flavor cigarettes (with the exception of menthol), require manufacturers of tobacco products to disclose the contents of their products, alterations made to their products, and research related to the effects of their products on the health of individuals who use their products [1]. Further, the regulations prohibit the use of descriptive terms such as "light", "mild", and "low tar" that have been used by the tobacco industry to market their products and have been demonstrated to be misleading to consumers [2]. However, the experience in other countries indicates the effects of such bans on smoker perceptions appears to be limited, at least in part because of the continued use of colors corresponding to old strength indicators [3,4].

The FSPTCA included a number of statutory deadlines for specific actions by the FDA:

January 2010: tobacco manufacturers and importers required submit information about ingredients and additives in tobacco.

July 2010: tobacco manufacturers prohibited from using the terms "light," "low," and "mild" on tobacco products.

July 2010: banned specific types of advertising and marketing including vending machines and self service displays in non adult only venues, branded product tie-ins (e.g., Marlboro t-shirts), free cigarette samples, outdoor advertising within 1000 feet of schools, event sponsorship, and tombstone only advertising in magazines and point of sale in non-adult only facilities and magazines with youth readerships. These actions related to advertising and marketing are currently stayed pending the outcome of litigation.

March 2011: Tobacco Products Scientific Advisory Committee completed mandated review on public health impact of menthol cigarettes, concluding that "removal of menthol cigarettes from the marketplace would benefit public health in the United States".

While the FSPTCA was landmark legislation for tobacco control in the United States, there is limited public opinion data assessing general attitudes toward FDA tobacco regulation. A June 2009 Gallup Poll indicated that 46% of all Americans and only 28% of smokers approved of FDA tobacco product regulation in concept [5]. The current manuscript reports data from a nationally representative sample of smokers measuring their attitudes and beliefs on specific issues relevant to the FSPTCA. Data were collected shortly after the passage of FSPTCA and prior to the enactment of any of the specific regulatory measures. Levels of support or opposition for specific regulations could be used by the FDA to inform methods for increasing consumer knowledge of tobacco related regulations.

## Methods

The data source for this study is the International Tobacco Control (ITC) United States Supplemental Survey. The ITC United States Survey--as all ITC Surveys being conducted across 20 countries--includes questions to assess smoking behavior, attempts at cessation, and attitudes and beliefs about tobacco products, as well as questions pertaining to each of the demand reduction policies of the WHO Framework Convention on Tobacco Control (FCTC) (e.g., warning labels, smoke-free laws, advertising/promotion, price/taxation) and a set of important psychosocial mediators and moderators of tobacco use and of cessation (e.g., perceived risk, quit intentions, time perspective). In general, the ITC Surveys are designed to evaluate the psychosocial and behavioral effects of national-level and sub-national tobacco control policies [6].

The ITC United States Survey began in 2002 and has been conducted approximately annually, in conjunction with ITC surveys in Canada, United Kingdom, and Australia; to date 8 waves have been conducted. The ITC United States Survey utilizes random digit dialing to recruit a sample of randomly selected adult (≥ 18 years) smokers. Cohort members who are lost to follow-up are replaced with newly recruited participants from the same sampling frame to preserve the overall sample size from wave to wave. Thus, there is a longitudinal component and a representative cross-sectional component at each wave of every ITC survey.

The ITC United States Supplemental Survey was conducted between November 2009 and January 2010 of the existing cohort at the previous wave of the ITC United States Survey (Wave 7, which had been conducted between October 2008 and July 2009).. This supplementary survey included the addition of focused questions around issues pertinent to the FSPTCA in order to measure baseline knowledge and attitudes about the FSPTCA around the time that the Act was passed. Further details of the sampling design used in the ITC survey can be found in a technical report, available on the ITC Project website at http://www.itcproject.org/[7].

The total eligible sample size for the 2009-10 survey was 912 participants who at the preceding wave of the ITC United States Survey (Wave 7) reported being a daily smoker of 10 or more cigarettes per day, reported that they regularly smoked a particular variety of cigarettes, and provided the type of location where they usually purchase their cigarettes. These inclusion criteria facilitated a component of the study where unopened cigarette packs were collected from participants (results not reported in this manuscript).

These data collection methods were reviewed and approved by the Roswell Park Cancer Institute Institutional Review Board and the University of Waterloo Human Research Ethics Committee.

Descriptive statistics were used to examine measures of general knowledge, attitudes, and beliefs regarding FDA regulation of tobacco products. In addition, a number of survey questions focused on smokers' beliefs regarding tobacco product descriptive terms such as light and mild and relationship these terms have with product safety. Bivariate chi-square test statistics were used to examine the relationship between various demographic measures (age, gender, race, income, education, and heaviness of smoking index (HSI) and the FSPTCA-relevant measures (see the tables for the exact question wordings). Confidence intervals (95%) for single population proportions were calculated for each point estimate reported in the manuscript and are presented within parentheses in each cell of the tables. Relatively few demographic differences were observed, which are noted in footnotes to each table. All analyses were conducted using SPSS 16.0 (Chicago, IL).

## Results

### Survey Response

A total of 678 of the 912 (74%) eligible smokers completed the supplemental survey.

### Knowledge and Beliefs About FDA Regulation

The results of a series of questions asking respondents about their knowledge and beliefs about FDA regulations, their components, and their implications are presented in table 1. Generally, most smokers interviewed were unaware of the pending FDA tobacco regulations as well as specific components. It is notable that nearly half think that the government has tested their brand and slightly more think their brand could be taken off the market due to safety concerns.

**Table 1:** Knowledge and Beliefs About FDA Regulation and Its Consequences (n = 678)

	% Yes	% No	% Don't Know
"In 2009, the President signed a law that gave the US Food and Drug Administration (FDA) power to regulate tobacco products. Have you heard of this law before?"	28* (24%-31%)	71 (68%-75%)	1 (1%-2%)

"If the government regulated tobacco products, would it suggest to you that they are more harmful than you think?"	43** (39%-47%)	52 (48%-56%)	5 (4%-7%)

"Has the government put rules in place designed to make cigarettes less harmful?"	25 (22%-28%)	61 (57%-65%)	14 (11%-17%)

"Do you think that such rules could make smoking less harmful?"	26*** (23%-30%)	65 (61%-68%)	9 (7%-11%)

"Do you think that tobacco companies disclose everything they know about cigarettes to the government?"	19 (16%-22%)	76 (73%-80%)	5 (3%-6%)

"Do you think your current brand of cigarettes has been evaluated by the government?"	43**** (39%-47%)	31 (27%-34%)	26 (23%-30%)

"Do you think the government could remove your brand of cigarettes from the market because of safety concerns?"	49 (45%-53%)	38 (34%-42%)	13 (10%-16%)

"Do you think that tobacco companies ARE NOW REQUIRED to disclose everything they know about cigarettes to the FDA?"	47 (43%-50%)	39 (36%-43%)	14 (11%-16%)

"Do you think that tobacco companies are sincere in their efforts to improve the health of their customers by providing them with information to help them quit?"	30 (27%-34%)	65 (61%-69%)	5 (3%-6%)

95% Confidence Intervals are presented in parentheses

* = compared to those with lower education, participants with higher education were more likely to report awareness of the law (p = .016)

** = compared to those with higher education, participants with lower education (p < .001) and lower income (p = .007) were more likely to report that if government regulated tobacco products, it would suggest that the products are more harmful

*** = compared to females, male participants were more likely to think rules designed to make cigarettes less harmful could make smoking less harmful (p = .032)

**** = compared to females, younger participants (18-24), and those with higher education, male participants (p = .008), older participants (p = .030), and participants with lower education (p = .039) were more likely to report a belief that their current brand of cigarettes has been evaluated by the government

### Support for Restrictions on Tobacco Product Marketing

As shown in Table 2, the majority of smokers indicated support for banning cigarette promotion (67%), but opposed the more specific proposal of requiring tobacco companies to sell cigarettes in plain packaging (only 24% were supportive). There was also only minority support (33%) for restrictions on the number of cigarette retail outlets (see table 3).

**Table 2:** Beliefs About Tobacco Company Marketing and Support for Restrictions (n = 678)

	% Agree	% Neutral	% Disagree	% Don't Know
"Tobacco companies should not be allowed to promote cigarettes at all, but merely make them available to adults who want to smoke them".	67* (64%-71%)	6 (4%-8%)	26 (23%-30%)	1 (0-1%)

"Tobacco companies should be required to sell cigarettes in plain packages -- that is, in packs without any brand names or fancy designs."	24 (20%-27%)	9 (7%-11%)	65 (61%-68%)	3 (1%-4%)

95% Confidence Intervals are presented in parentheses

* = compared to older participants, younger participants (18-24) were more likely to agree (p = .003)

**Table 3:** Beliefs Regarding Restrictions on Tobacco Retail Outlets (n = 678)

	% Support	% Oppose	% Don't Know
"Would you support or oppose a law that restricted the number of places where cigarettes could be purchased?"	33 (29%-36%)	65 (61%-68%)	2 (1%-3%)

95% Confidence Intervals are presented in parentheses

### Beliefs Regarding Additives, Flavorings, and Menthol

As shown in Table 4, 41% of smokers supported a law that would ban additives and flavorings that make cigarettes seem less harsh. However, when asked about a more specific proposal to ban menthol favoring in cigarettes, only 19% supported it. A comparison of menthol and non-menthol cigarette smokers indicated a statistically significant difference with respect to support for banning menthol (9% vs. 22%, respectively; p < .001).

**Table 4:** Support for Banning Additives, Flavorings, and Menthol (n = 678)

	% Support	% Oppose	% Don't Know
"Would you support or oppose a law that banned additives and flavorings that make cigarettes seem less harsh?"	41* (37%-45%)	55 (51%-59%)	4 (3%-6%)

"Would you support or oppose a law that banned menthol cigarettes?"	19 (16%-22%)	75 (72%-79%)	6 (4%-7%)

95% Confidence Intervals are presented in parentheses

* = compared to female participants, male participants were more likely to support (p = .034)

### Beliefs Related to Regulations that Limit Nicotine, Cigarettes, and Tobacco Products

Smokers were asked three questions about their opinions and beliefs about potential policies ranging from reducing the addictiveness of tobacco to banning tobacco products altogether if nicotine were made available in some other non-cigarette form. The results are presented in table 5. There were high levels of support for decreasing the addictiveness of cigarettes (67%). Although support was much lower for the other two potential policies, it is notable that significant minorities of smokers did express support for banning cigarettes (30%) and for banning tobacco (19%) if alternative sources of nicotine were available.

**Table 5:** Beliefs Related to Regulations that Limit Nicotine, Cigarettes, and Tobacco Products (n = 678)

	% Support	% Oppose	% Don't Know
"If nicotine was made easily available in non-cigarette form, would you support or oppose a law that reduced the amount of nicotine in cigarettes, to make cigarettes less addictive?"	67* (64%-71%)	28 (25%-31%)	5 (3%-7%)

"If nicotine was made easily available in non-cigarette form, would you support or oppose a law that banned cigarettes but made alternative forms of nicotine available?"	30 (26%-33%)	66 (63%-70%)	4 (3%-6%)

"If nicotine was made easily available in non-cigarette form, would you support or oppose a law that banned tobacco products completely?"	19** (16%-22%)	79 (76%-82%)	2 (1%-3%)

95% Confidence Intervals are presented in parentheses

* = compared to female participants, male participants were more likely to support (p = .001)

** = compared to high and moderate income participants, low income participants were more likely to support (p = .004)

### Beliefs About "Light" and "Low Tar" Cigarettes

Misperceptions about "light" and "low tar" cigarettes were evident in the responses from many smokers. These results are presented in table 6. Twenty percent reported that light cigarettes are less harmful than regular cigarettes and 24% said that light smokers take in less tar compared to those smoking full strength cigarettes.

**Table 6:** Beliefs About Light and Mild Descriptors (n = 678)

	% Agree	% Neutral	% Disagree	% Don't Know
"Light cigarettes are less harmful than regular strength cigarettes."	20 (17%-23%)	7 (5%-9%)	70* (67%-74%)	3 (1%-4%)

"Smokers of light cigarettes take in less tar than smokers of regular strength cigarettes."	24 (21%-27%)	10 (8%-12%)	59** (55%-62%)	7 (5%-9%)

95% Confidence Intervals are presented in parentheses

* = compared to older participants, younger participants (18-24) were more likely to disagree (p < .001)

** = compared to older participants, younger participants (18-24) were more likely to disagree (p = .031)

## Discussion

The results of this study show that most adult smokers were unaware of the FDA's authority to regulate tobacco products, but were generally supportive of efforts to better inform the public about health risks and to make tobacco products less addictive.

These findings from a nationally representative sample of smokers provide an indication of support for the specific provisions of the FSPTCA. The study is limited since it only includes adult smokers and because this supplemental survey wave was conducted shortly after the passage of FSPTCA and prior to the implementation of any of the components of the regulations. Thus, it is not surprising that awareness of these new regulatory measures was low. Future waves of the ITC United States Survey will continue to monitor how smokers' beliefs and attitudes related to FDA regulation of tobacco products may change over time.

Support for potential regulations foreshadowed in the FDA legislation was generally stronger than for some of the more novel possibilities. Support for many FDA proposals was consistent among all smoker subgroups examined, including heavy smokers. Of the small number of statistically significant comparisons, most were related to age. When compared to older participants, 18-24 year olds were less likely to believe that their brand has been evaluated by the government, more likely to support a ban on tobacco company promotions, and were more likely to believe that light cigarettes are just as dangerous as regular strength cigarettes. This suggests a need to better target educational campaigns toward older smokers.

The finding that support is generally strong among heavy smokers, and comparable to that of light smokers, could be particularly important because it suggests that the degree of nicotine dependence is not motivating opposition to regulation.

For some measures there was markedly less support for specific policies than for the general proposition under which the specific proposal logically falls. For example, there was much stronger support for removing promotional activities than the specific measure of requiring cigarettes to be sold in only plain packaging. In these cases, it is important to understand whether this is because smokers do not believe the policy will achieve the general aim or whether they think it is an inappropriate means of achieving it.

Strong support was found among smokers for banning cigarette promotion as long as adults who want to smoke could still purchase cigarettes. Smokers also indicated some level of support (33%) for restricting the number of places where cigarettes could be sold.

Support for banning additives and flavorings was generally low. About one-fifth of current smokers (19%) and 9% of current menthol smokers supported banning menthol. Support for banning menthol was unrelated to interest in quitting, which may suggest that smokers don't link flavoring and ease of quitting. These findings are similar to another nationally representative survey conducted in November 2009. Respondents were asked whether they strongly agree, agree, disagree, or strongly disagree with the statement: "Menthol cigarettes should be prohibited just like other flavored cigarettes." Winickoff et al. found that 28% of current smokers supported banning menthol cigarettes [8].

Data from this study suggest that smokers are supportive of actions that they see as better enabling them to make free choices about the use of cigarettes. Nicotine addiction and a lack of knowledge about these products are two critical constraints now placed on consumers that limit their ability to freely choose when and how to use cigarettes. That said, they still want the experience of smoking to be as pleasant as possible, something not consistent with this desire.

The data presented here show that smokers are generally supportive of decreasing the addictiveness of the product and the level of support is dependent on the array of alternatives that were offered to them. Few supported banning cigarettes or tobacco products altogether, an action not permitted under the FSPTCA. Given that approximately 90% of smokers regret smoking and most want to quit [9], reducing nicotine levels in cigarettes has the potential to make it easier for smokers to quit. These data suggest that reducing nicotine levels to make cigarettes less addictive is a potentially viable strategy the FDA can take to improve public health. However, we do not know how smokers would respond to a specific measure where they might have a stronger sense of how the regulation would impact them directly.

It is clear from this study that smokers need to be better informed about the implications of FDA regulation. A large scale public education campaign from the FDA about the dangers of tobacco use and the regulatory powers they now have that is coordinated with other national, state, and local partners would be useful. Because reducing nicotine levels has the potential to reduce addictiveness, thereby increasing consumer autonomy, such efforts to educate the public about this possible regulatory action would be important.

These baseline data were collected shortly after the passage of the FSPTCA and prior to the enactment of any specific regulatory measures. These initial levels of support or opposition for specific policy measures can be used by the FDA to inform policy development and the need to educate smokers and the public at large about the purpose behind the regulation and of the kinds of regulatory controls the public are looking for. As specific regulatory measures of the FSPTCA are enacted, it will be important to assess any changes in knowledge and attitudes related to specific components of the regulations.

## Competing interests

RJO has served as a consultant to the Tobacco Products Scientific Advisory Committee (Tobacco Constituents Subcommittee) of the Food and Drug Administration. Otherwise, the authors have no competing interests to declare.

## Authors' contributions

RJO, GTF, RB, KMC, AH: conception and survey design. BVF: data analysis. BVF, RJO, GTF, RB, KMC, AH: drafting the manuscript and revising it critically for important intellectual content. All authors read and approved the final manuscript.

## Pre-publication history

The pre-publication history for this paper can be accessed here:

http://www.biomedcentral.com/1471-2458/11/941/prepub
